# Enhanced detection of oral dysplasia by structured illumination fluorescence lifetime imaging microscopy

**DOI:** 10.1038/s41598-021-84552-8

**Published:** 2021-03-02

**Authors:** Taylor A. Hinsdale, Bilal H. Malik, Shuna Cheng, Oscar R. Benavides, Maryellen L. Giger, John M. Wright, Paras B. Patel, Javier A. Jo, Kristen C. Maitland

**Affiliations:** 1 Department of Biomedical Engineering, Texas A&M University, College Station, USA; 2 Department of Radiology, The University of Chicago, Chicago, USA; 3 Department of Diagnostic Science, Texas A&M College of Dentistry, Dallas, USA; 4 Present Address: Delft University of Technology, Delft, The Netherlands; 5 Present Address: QT Imaging, Inc, 3 Hamilton Landing, Suite 160, Novato, CA 94949 USA; 6 Present Address: Department of Electrical and Computer Engineering, University of Oklahoma, Norman, USA

**Keywords:** Oral cancer, Applied optics, Microscopy, Oral cancer, Fluorescence imaging

## Abstract

We demonstrate that structured illumination microscopy has the potential to enhance fluorescence lifetime imaging microscopy (FLIM) as an early detection method for oral squamous cell carcinoma. FLIM can be used to monitor or detect changes in the fluorescence lifetime of metabolic cofactors (e.g. NADH and FAD) associated with the onset of carcinogenesis. However, out of focus fluorescence often interferes with this lifetime measurement. Structured illumination fluorescence lifetime imaging (SI-FLIM) addresses this by providing depth-resolved lifetime measurements, and applied to oral mucosa, can localize the collected signal to the epithelium. In this study, the hamster model of oral carcinogenesis was used to evaluate SI-FLIM in premalignant and malignant oral mucosa. Cheek pouches were imaged in vivo and correlated to histopathological diagnoses. The potential of NADH fluorescence signal and lifetime, as measured by widefield FLIM and SI-FLIM, to differentiate dysplasia (pre-malignancy) from normal tissue was evaluated. ROC analysis was carried out with the task of discriminating between normal tissue and mild dysplasia, when changes in fluorescence characteristics are localized to the epithelium only. The results demonstrate that SI-FLIM (AUC = 0.83) is a significantly better (p-value = 0.031) marker for mild dysplasia when compared to widefield FLIM (AUC = 0.63).

## Introduction

Oral squamous cell carcinoma (OSCC) is commonly characterized by a poor prognosis and a low 5-year survival rate of 65%^1, 2^. Prognostic outcomes can be improved by early detection of the disease, however, nearly 70% of all oral cancers are diagnosed in advanced disease stages where the 5-year survival rate drops dramatically to 39%^1^. As with most cancers, a variety of environmental and genetic conditions can lead to its development. Some main risk factors associated with the development of OSCC in humans are the usage of tobacco and alcohol, especially in conjunction; the human papilloma virus (HPV); and genetic predisposition to developing the disease^2, 3^. Oral cancer is of particular concern because of its propensity to cause either death or greatly diminish the quality of life for patients who survive diagnosis and treatment^4^.

The current standard to screen for oral cancer is via a visual examination of the oral cavity by a trained clinician with subsequent tissue removal and histopathological analysis, if a suspicious lesion is identified^5^. The gold standard has superior reliability as a diagnostic method, however, the ability to discriminate between benign and premalignant lesions much sooner in the disease progression is necessary to improve the early detection rate. Several studies have shown that experienced clinicians often struggle to differentiate premalignant from benign oral lesions^6, 7^. There are two major problems with the current screening method: first is the inherent time delay between the identification of a potentially malignant lesion and its accurate histopathological diagnoses; second is that suspicious oral cancer lesions are often diffuse and multi-focal, which makes screening the oral cavity even more challenging.

There are currently several commercially available screening tools for oral cancer, such as vital tissue staining, brush cytology, and saliva testing. Various optical imaging and sensing methods have also been developed and studied for oral cancer diagnosis, and these include, but are not limited to, narrow band imaging, fluorescence and reflectance confocal microscopy, optical coherence tomography, and widefield autofluorescence microscopy^8–14^. Unfortunately, none of these imaging or non-imaging techniques have yet to become an accepted standard replacement for or adjunct to the conventional screening methods^15, 16^. There is a tremendous need for a screening tool that can accurately and quickly diagnose malignant and premalignant lesions within the mouth.

Nicotinamide adenine dinucleotide (NADH) and flavin adenine dinucleotide (FAD) are metabolic cofactors that have shown potential as endogenous biomarkers of precancer and cancer progression^17–19^. During carcinogenesis, the fundamental cellular mechanisms for metabolism begin to shift from oxidative phosphorylation to glycolysis^17^. This causes a shift in the cellular microenvironment of NADH and FAD that contributes to a change in their fluorescence lifetimes. Most of these changes occur in the metabolically active epithelium, which is where most OSCCs originate, rather than the submucosa^17–21^. Fluorescence lifetime imaging microscopy (FLIM) is a label-free metabolic imaging modality that is sensitive to variations in local microenvironmental factors, such as pH, temperature, and protein binding, and can be used to deduce malignancy, or lack thereof of, in tissue. Collagen is also affected by the onset of carcinogenesis, and the reduction in fluorescence levels due to collagen crosslink degradation is detectable using FLIM^22^. Reduction in collagen fluorescence is also attributed to inflammatory conditions confounding specificity of diagnostic devices that are dominated by collection of stromal fluorescence. Our group has recently shown that these fluorescent markers can be used to differentiate between oral lesions of low-risk and high-risk for malignancy in a hamster cheek pouch model with 87% sensitivity and 94% specificity^19^. Of greater diagnostic value to the clinician would, however, be the ability to distinguish between benign and premalignant lesions earlier in the disease progression.

FLIM can be realized utilizing a vast array of measurement techniques, e.g. time correlated single photon counting (TCSPC), direct decay measurement, and intensified CCD cameras (ICCD). An ICCD can capture all pixels within the field of view in parallel, and with the advent of the rapid lifetime determination method, it can potentially be used to easily image dynamic physiological environments, a prerequisite for in vivo imaging^23^. While FLIM offers an exceptional ability to distinguish between different fluorophores within the focal plane of the imaging system, it suffers from the inability to accurately resolve multiple spectrally overlapping lifetimes that exist within a single pixel. This can be attributed to either multiple elements inhabiting the same pixel in the focal plane or out of focus fluorescence from different focal planes summing inside the pixel of interest. Multi-exponential or stretched exponential fitting algorithms can be used to determine the distribution of lifetimes present in a pixel, but implementing these methods in a widefield configuration using an ICCD would require more than two gates and would significantly slow the process^24, 25^. Although not much can be done to alleviate the occupancy of a single pixel by multiple fluorophores without the use of multiple exponentials, optical sectioning techniques can be employed to diminish the impact of out of focus and spectrally overlapping fluorescence^26–28^. While optical sectioning techniques such as confocal FLIM may be prohibitively slow for clinical imaging, structured illumination microscopy (SIM) has been used and combined with FLIM to accurately recover fluorescence lifetimes at reasonable acquisition rates^26^. More recently, SIM and widefield FLIM were combined and used to acquire super-resolved images of cell morphology and map the corresponding Förster resonant energy transfer (FRET) readouts to the cellular nanostructures^29^.

The aims of the present study were to investigate structured illumination fluorescence lifetime imaging microscopy (SI-FLIM) as a screening method for early stages of premalignant lesions, and to show that SI-FLIM is superior in this regard when compared to widefield FLIM on its own. Note that the previously reported SI-FLIM technique was not applied to thick samples to determine whether accurate lifetimes could be recovered from samples with large overlapping layers that contain different fluorophores^26^. Our group had previously shown that not only can SIM be used in epithelial tissue imaging, but that SI-FLIM can be used to image relatively thick tissue models and epithelial tissue^30, 31^.

## Materials and methods

### Tissue preparation

The Syrian golden hamster (*Cricetus auratus*) model for OSCC using 7, 12-dimethlybenzene[a]anthracene (DMBA) was used because its application to the hamster model of sequential oral oncogenesis closely resembles the formation of oral cancer in humans^2, 19^. The animal use protocol (AUP# 2014-0230) was approved by the Texas A&M University Institutional Animal Care and Use Committee (IACUC). All animals were cared for in accordance with the National Institutes of Health Public Health Service Policy on Humane Care and Use of Laboratory Animals. All experiments were conducted according to ARRIVE guidelines. Twelve male hamsters were acquired between five and six weeks in age and were allowed to acclimate to their environment for one week. The hamsters were randomly divided into four groups of three hamsters, a control group and three treatment groups. The treatment groups represented six-week, nine-week, and twelve-week treatments with DMBA. The animals in the treatment groups received an application of DMBA as the carcinogenic factor in mineral oil (as a carrier) on the right cheek pouch and pure mineral oil on the left cheek pouch. The control group exclusively received application of pure mineral oil. Treatments were applied to the hamsters under 3–4% isoflurane anesthesia. The mineral oil solutions were administered via No. 5 paint brushes. Hamsters were treated three times per week for the duration of their treatment periods. Beginning at the sixth week of the treatments, the three control hamsters were imaged and euthanized along with the sixth-week treatment group. At the designated time points, the ninth-week group and twelfth-week group were imaged and euthanized accordingly. The cheek pouch tissues were harvested post-mortem for histopathological processing.

### Imaging protocol

**Figure 1:**
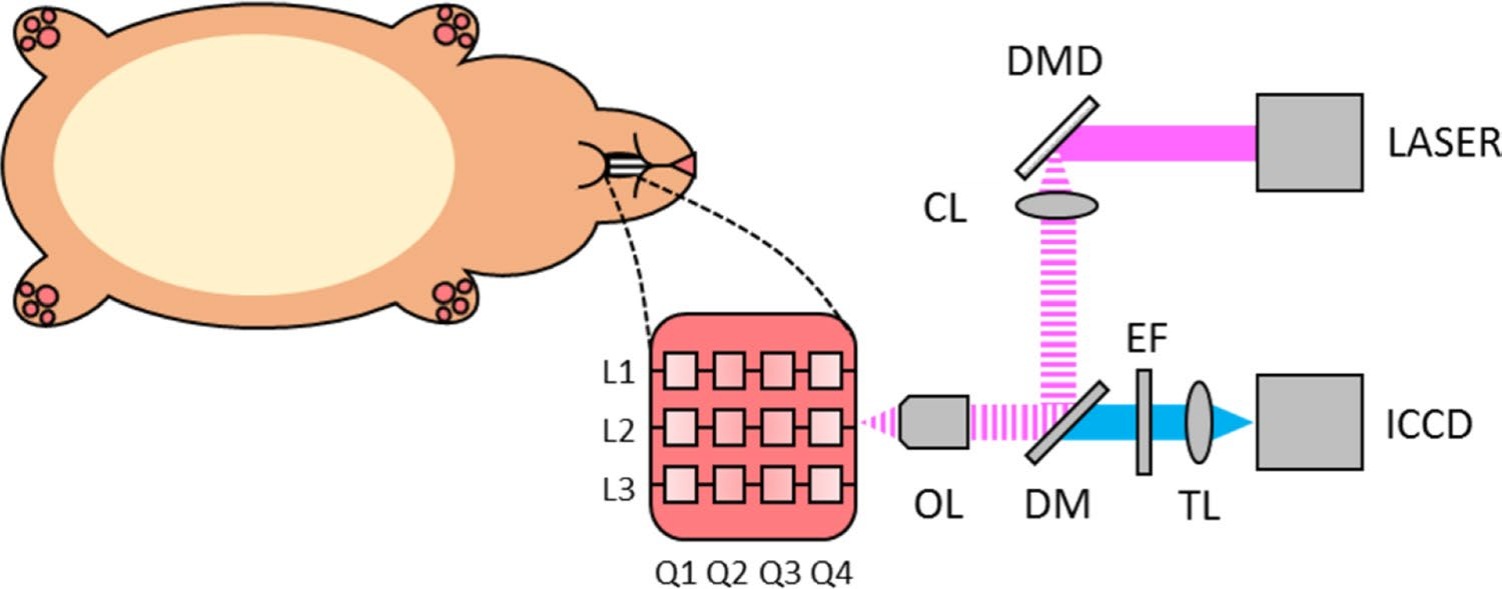
Diagram of experimental setup for imaging oral carcinoma development in hamster cheek pouch. A hamster is anesthetized and its cheek pouch is everted to give ~ 16 mm × 16 mm imaging area. Imaging was performed at twelve sites located in three lines (L1, L2, L3) divided into four quadrants (Q1, Q2, Q3, Q4). A conceptual drawing of the SI-FLIM devices is shown on the right. OL (objective lens), DM (dichroic mirror), EF (emission filter), TL (tube lens), ICCD (intensified CCD), CL (collimation lens), DMD (digital micromirror device), Laser (355 nm pulsed laser).

Immediately prior to imaging, each hamster was anesthetized with a 10% w/v urethane (Sigma-Aldrich) solution in de-ionized water. After the animals became fully sedated, one cheek pouch was mounted into a custom-made brace designed to immobilize and flatten the tissue, yet allow blood flow to the cheek. This process was performed on the treated pouch and the mineral-oil-only contralateral pouch separately. The exposed cheek area available for imaging, approximately 16 mm × 16 mm, was subdivided into three imaging lines that were equally spaced along one of the 16 mm dimensions to facilitate histology registration. These three lines were further divided into four equally spaced quadrants, resulting in a 4 × 3 grid with 12 defined imaging regions as shown in Fig. 1. Any regions of the epithelium that appeared visually distinct to the eye were also imaged separately after the first twelve images. For the contralateral side, five images were taken equally spaced over the exposed area.

The previously reported SI-FLIM system combines metabolic sensitivity of FLIM with optical sectioning of structured illumination to probe layers of tissue^31^. Briefly, the illumination path projected a 10 kHz 1.5 ns pulsed 355 nm laser (SPOT-10-100-355, ELFORLIGHT) onto a digital micromirror device (DMD, DLi D4100, DLi). The DMD was programed to display either a modulation pattern (structured illumination) or no pattern (widefield). The DMD was then imaged onto the sample plane by a 20X objective lens (N20X-PF, ThorLabs). The modulation pattern for structured illumination was 24 pixels per line, resulting in a modulation frequency of 30.45 lines per mm at the sample. The autofluorescence from the sample was then collected and imaged with an intensified charge-coupled device (ICCD, 4Picos, Stanford Computer Optics) that was synchronized with the pulsed laser. The optical sectioning full-width half-maximum was approximately 35 μm for structured illumination. Positioning the superficial layer of tissue within the focal plane facilitates signal collection from this layer and suppression of autofluorescence from stromal collagen.

To measure lifetimes on a widefield camera, we utilized the rapid lifetime determination (RLD) method, where capturing a standard widefield FLIM image requires a minimum of two time-gated images per lifetime^32^. The method requires that the fluorescence lifetime be integrated over a minimum of two time-gates so that a relationship between the integrated intensities and the fluorescence decay can be made. This is done by synchronizing an ICCD, with picosecond gating capabilities, to a 1 ns pulsed laser. The first image is taken with zero delay relative to the fluorescence emission decay after the arrival of a laser pulse. A second gated image is then taken with a delay equal to half of fluorescence lifetime of interest. In general, each gate width is equal to half of the fluorescence lifetime and many gates are averaged over a single CCD exposure. The ratio of intensities between the first and second gates can be used in Eq. (2) to approximate lifetime of the decaying fluorophore. However, to perform SIM, a minimum of six gated images, three modulated phase shifted images per time delay, are required^31^. Three spectral emission bands were imaged: 390 ± 20 nm, 450 ± 20 nm, and 550 ± 16 nm. These three bands correspond to the primary spectral band of the emission of the three fluorescent markers of carcinogenesis: collagen, NADH, and FAD, respectively. Figure 1 shows a diagram of the imaging setup and how it was used on the hamster cheek. For future context, when referring to the spectral channel being imaged, we will refer to them by the expected dominant fluorophore of that specific bandwidth. To remain consistent, the imaging order of spectral bands was maintained as 1) NADH, 2) FAD, and 3) collagen. Each channel was imaged with WF-FLIM. Only the NADH channel was measured with SI-FLIM first then WF-FLIM second. Moving forward, the NADH channel as measured by SI-FLIM will be labeled as SI-NADH, and the NADH channel as measured by widefield FLIM will be labeled as WF-NADH.

### Image processing

The FLIM data were obtained in two formats: raw intensity images for each widefield gate and raw modulated phase-shifted images for each SIM gate. The raw structured illumination images were first processed using the standard three-phase demodulation technique to create sectioned images for each gate delay^33^. Equation 1 was used to calculate the sectioned images where I_SIM_ is the sectioned image and I_1_, I_2_, and I_3_ are the modulated phase shifted images.

1 $${\text{I}}_{{{\text{SIM}}}} = \sqrt {({\text{I}}_{1} - {\text{I}}_{2} )^{2} + ({\text{I}}_{2} - {\text{I}}_{3} )^{2} + ({\text{I}}_{1} - {\text{I}}_{3} )^{2} }$$

A widefield image can be calculated by averaging the three phase-shifted gates and was used for SNR calculations. The sectioned images along with their widefield counterparts were then used to calculate fluorescence lifetime maps for each spectral band using the rapid lifetime determination method shown in Eq. (2), where τ is the fluorescence lifetime, Δt is the width and delay of the intensity gates, and D_1_ and D_2_ are the first and second gated images, respectively.

2 $$\uptau = \frac{{ - \Delta {\text{t}}}}{{\ln \left( {\frac{{{\text{D}}_{2} }}{{{\text{D}}_{1} }}} \right)}}$$

Note that the width and the delay of the intensity gates are the same size for simplicity; however, this is not a strict requirement of the acquisition. Fundamentally, Eq. (2) is taking the integrated intensity under the fluorescence decay and creating a ratio from both gates. This method is quick and easily implemented when calcualting average lifetime values from images captured by an ICCD. Before computing the fluorescence lifetime images using Eq. (2), image masks were generated so that only pixels of sufficient quality were included in our final lifetime analysis. The first mask was based on gray level thresholding to distinguish in focus from out of focus regions using the sectioned intensity images. The other mask was based on signal to noise ratio (SNR) and utilized a sliding SNR filter to calculate SNR maps. Here, the signal is defined as the mean of a sliding 7 × 7 kernel and the noise is the standard deviation of the background. The SNR mask was based on the second gated image for the widefield and sectioned modalities. The second gate was used to calculate SNR maps to ensure that the lowest intensity acted as the cutoff limit. The first set of SNR masks were generated using the standard widefield intensity image and the reconstructed widefield intensity image. The SNR cutoff for each mask was set to > 20 dB, a typical value for fluorescence lifetime imaging^34, 35^. The third SNR mask was made using the second gate of the sectioned images. Structured illumination is known to have relatively reduced SNR when compared to widefield. Because of this, we lowered our SNR criteria for the sectioned image to > 13 dB^34, 36^. Even though this value is lower than desirable and future systems would take great care to enhance the SNR, it is sufficient. The lifetime images were then calculated using Eq. (2) and spatially averaged using a smoothing mean filter. A histogram was then made of each lifetime image and the median value was extracted for statistical analysis. The median values are useful for visualizing trends within an animal, but lack robustness when it comes to cross animal comparison. This could be due in part to physiological conditions that developed in individual hamsters over the course of treatment unrelated to carcinogenesis. To normalize for this, the median lifetime values from the contralateral, mineral oil only, images were averaged on a per animal basis and subtracted from the images on their paired treatment sides. In this way, a lifetime delta was created between the treatment and contralateral side and helped reduce inter-subject variability by providing a lifetime shift from a normal baseline for every animal. The contralateral normalization was done for both WF-NADH and SI-NADH independently from one another.

### Statistical analysis

A statistical analysis was carried out on the normalized median lifetime values that were extracted from each image. To perform a comparison of the values, they were put into three main classes based on histopathology: normal, mild dysplasia, and carcinoma. The three classes contained 26, 17, and 9 regions of interest (ROIs), respectively. The classes were based on histopathological diagnoses provided by an experienced clinical pathologist (JW) viewing hematoxylin and eosin stained sections on a pathology microscope. Histology sections were cut along lines L1, L2, L3, and diagnoses were made for locations corresponding to each of four quadrants Q1, Q2, Q3, Q4 (Fig. 1). Each class (normal, mild dysplasia, and carcinoma) spans several hamsters as well as contains multiple samples from the same animal. As mentioned in the previous section, a normalization procedure was done to reduce the effects of inter-subject variability. For further analysis, it is relevant to note that although some samples’ ROIs were in close proximity to one another and used the same histopathological classification, none of them were physically overlapping. This allows us to treat each ROI as an independent data point. To start, each class was tested for normality and followed by two one-way ANOVAs. The two one-way ANOVAs were run to compare the separation of the normal, mild dysplasia, and carcinoma classes for each detection method. Due to unequal variances, the Brown-Forsythe method was used for the initial ANOVA testing^37^. After significance was found, a post-hoc analysis was conducted using the Games-Howell correction for unequal variance and sample size^38^. We then generated ROC curves using a proper binormal model in the task for distinguishing mild dysplasia from normal tissue, using the area under the ROC curve (AUC) as the performance metric^39^. This was done to compare and contrast SI-NADH against WF-NADH with the ultimate goal of showing that SI-FLIM has the potential to be a better indicator of the early stages of pre-malignancy when compared to widefield FLIM.

## Results

### The effects of SI-FLIM on the measurement of the NADH channel

**Figure 2:**
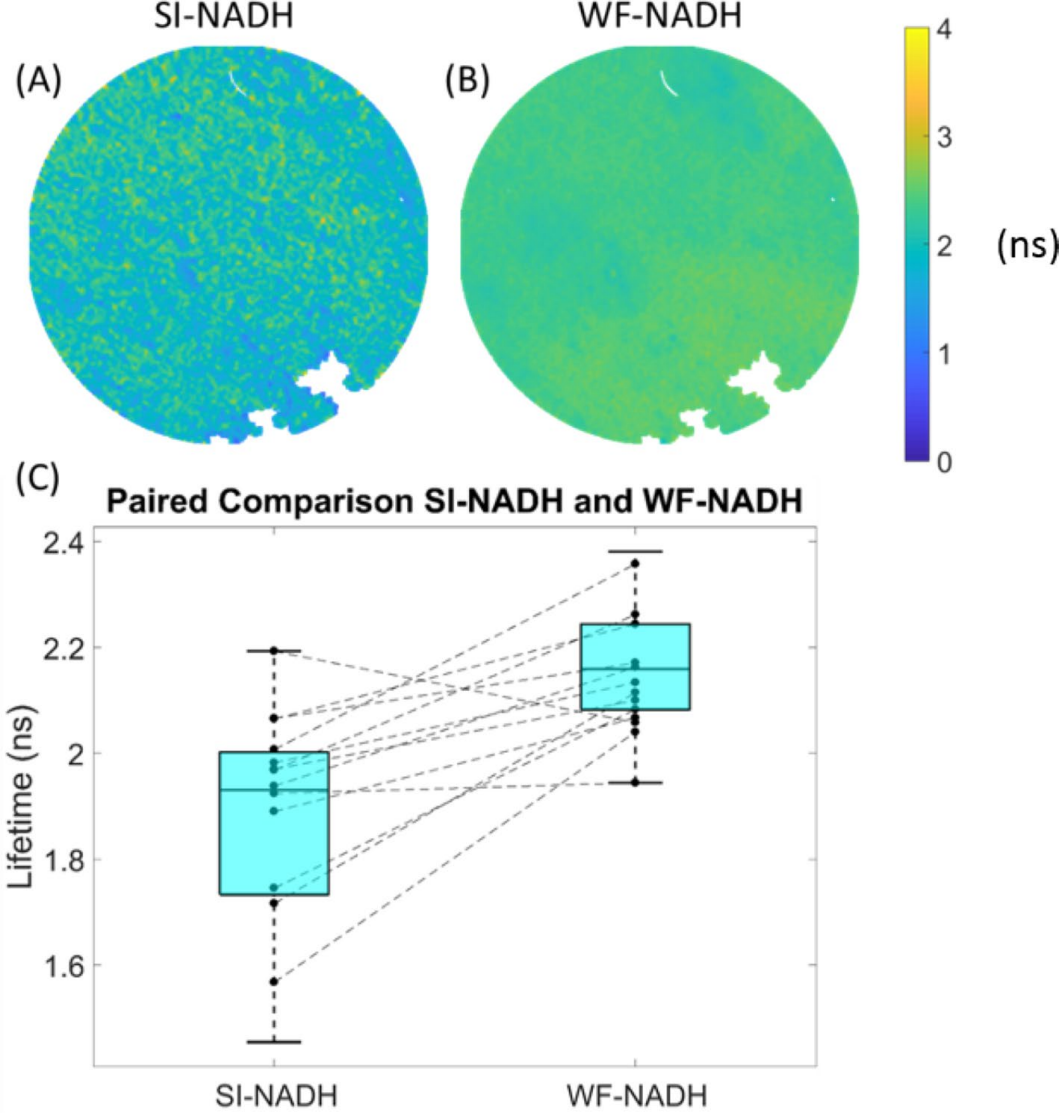
(**A**) SI-NADH image and (**B**) WF-NADH image of an example control hamster cheek pouch. (**C**) Paired relationship plot between SI-NADH and WF-NADH for control animals. SI-NADH on the left is shown to be generally lower than WF-NADH on the right. A paired t-test revealed that the observed relationship in the graph is statistically significant.

Our initial hypothesis was that SI-FLIM would be better able to reconstruct the true NADH fluorescence when compared to widefield FLIM. As discussed earlier, the hypothesized reason behind this is a rejection of the background fluorescence emanating from the collagen channel that is spectrally overlapped with the foreground NADH channel. Figure 2 illustrates the effects of SI-FLIM on the measurement of the NADH channel quite clearly. Note the data compared in Fig. 2 is from before the normalization procedure. Figure 2A,B represent the SI-NADH and WF-NADH lifetime values for a normal control hamster. To illustrate the distributions and paired comparisons of the WF-NADH and SI-NADH channels for the control hamsters, every other data point (to facilitate clear visualization of paired data) is plotted in Fig. 2C. To statistically compare SI-NADH against WF-NADH, a paired t-test was performed on all of the control hamster data points and provided a significant difference between the two groups (*p* < 0.05).

### Typical data of SI-FLIM

**Figure 3:**
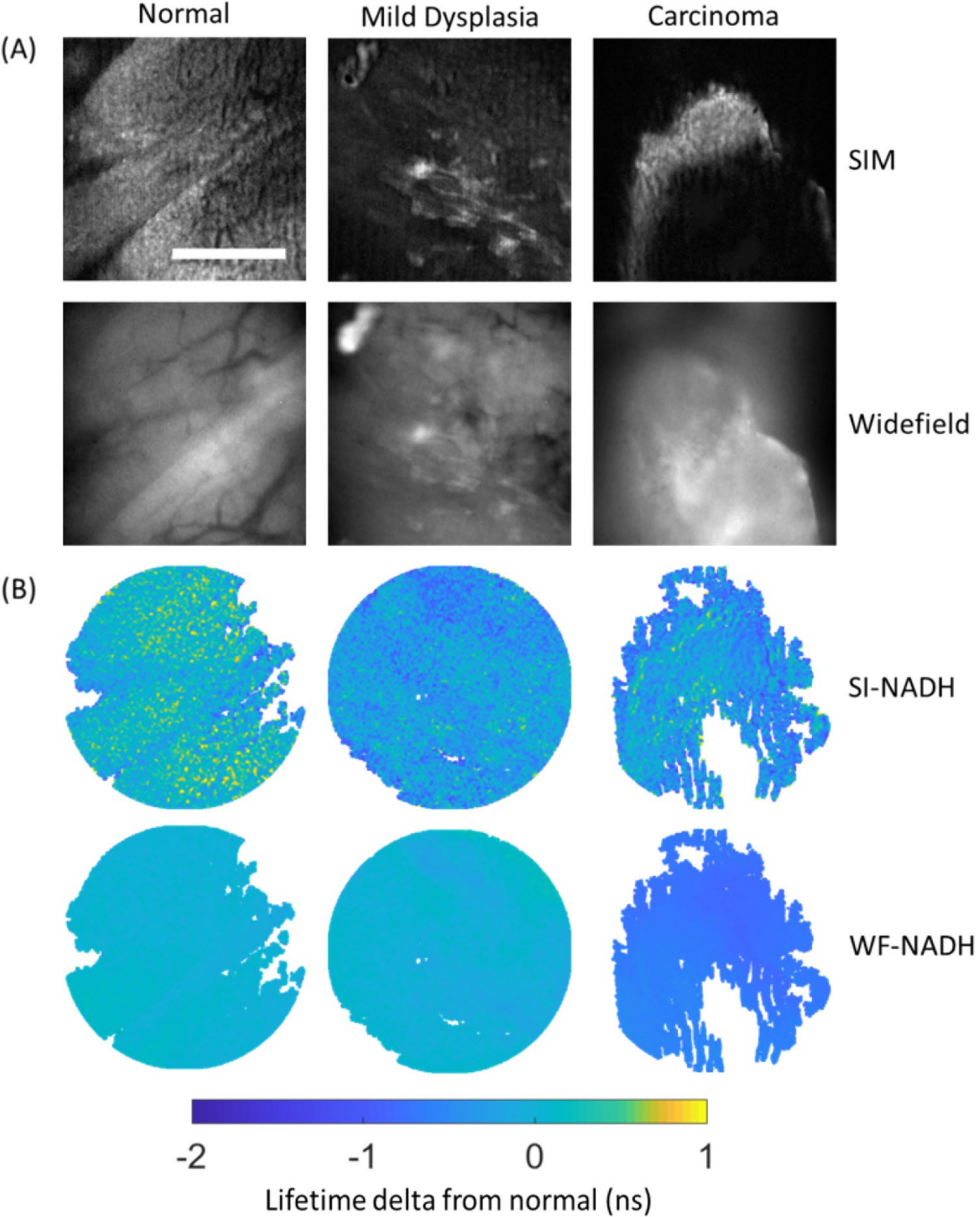
Example structured illumination and widefield image dataset of (**A**) fluorescence intensity and (**B**) fluorescence lifetime images for (left) normal, (center) mild dysplasia, and (right) carcinoma. Fluorescence lifetime scale is presented as delta change from the median lifetime obtained from paired contralateral normal images. Scale bar is 220 µm in length.

Before exploring the results, it is helpful to glimpse a snapshot of a typical SI-FLIM data set. For all regions of interest, a typical SI-FLIM data set consists of a series of time-gated images from both structured illumination, SI-FLIM, and WF-FLIM. These images are then used to compute the lifetime maps that accompany the gated images in Fig. 3. However, only the NADH channel is imaged with both modalities, while FAD and collagen are only imaged with widefield FLIM. For this reason, we will focus on presenting the differences between the SI-NADH and WF-NADH channels. As discussed earlier, all lifetime values displayed in Fig. 3 are the change in lifetime from the contralateral lifetime values of each channel (lifetime delta from normal). The lifetime images are circularly cropped to the smallest dimension of the detector plane.

The lifetime delta values become lower relative to the normal cases for both imaging modalities with the progression of carcinogenesis. This can be attributed to a shift of the fundamental metabolic processes of the epithelial cells from an oxidative metabolic state to a glycolytic metabolic state that results in a shortening of the NADH lifetime^18^. Therefore, on average, the lifetime delta should become more negative as cases become more severe. Slight lifetime delta variation observed in the SI-NADH images of normal tissue can be attributed to three main factors. First, since SI-FLIM is an optical sectioning method, the contribution of signal is expected to vary more significantly across the imaging field; whereas, with WF-FLIM, this variation is blurred out or lost. The second factor is the effect of undulations or variations of the tissue surface or layers of tissue, also due to the optical sectioning of SI-FLIM. Finally, additive variance from subtracting phase-shifted images results in an increased lifetime variance in the final images. Nevertheless, this overall lifetime delta variation between SI and WF is a small amount as detailed in the next section, but spatial variation in lifetime delta is observable with SI-FLIM.

### Statistical comparisons

Brown-Forsythe ANOVA tests performed on the normal, mild dysplasia, and carcinoma classes for the WF-NADH channel yielded statistically significant results (*p* = 0.007). Similarly, the Brown-Forsythe test for the SI-NADH channel yielded a *p* value = 0.001. Consequently, post-hoc tests utilizing the Games-Howell correction were performed. Note that the post-hoc tests were only comparing the classes within a specific channel, i.e. WF-NADH (mild dysplasia = carcinoma, mild dysplasia = normal, normal = carcinoma).

**Figure 4:**
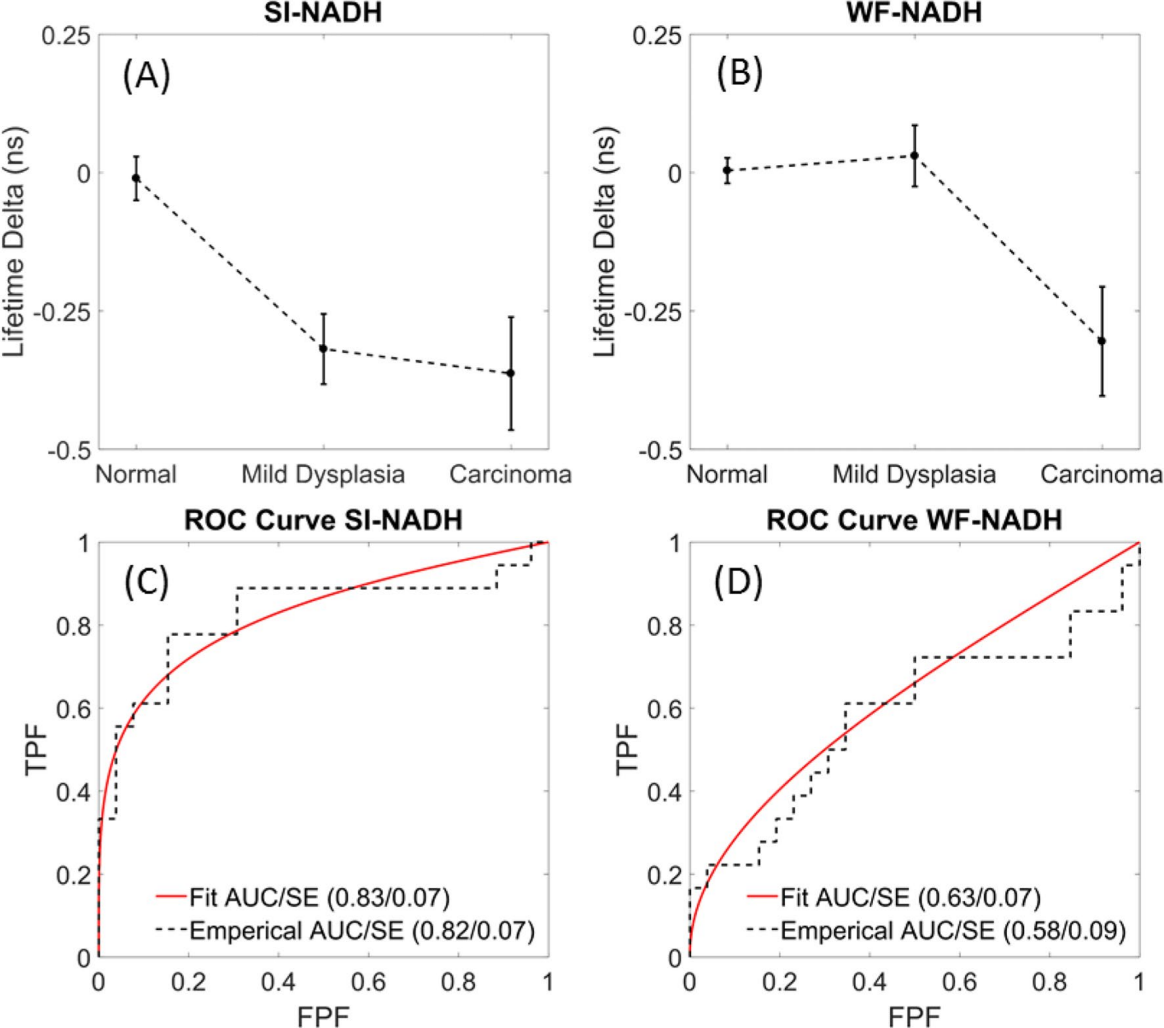
Trend in lifetime deltas of the (**A**) SI-NADH and (**B**) WF-NADH channels for normal, mild dysplasia, and carcinoma. Data points are of the mean lifetime delta and error bars are ± one standard error. ROC curves compare the differentiating power of (**C**) SI-NADH and (**D**) WF-NADH for cases of mild dysplasia against normal. The fitted curves were made using a proper binormal model.

Table 1 indicates that SI-NADH discriminates between normal tissue and mild dysplasia with high statistical significance (*p* = 0.001); whereas, WF-NADH was unable to detect a difference (*p* = 0.897) between normal and mild dysplasia. This is important in clinical cases where the early identification of pre-malignant lesions is critical to patient health enabling follow up monitoring and repeated histologic assessment, if warranted, to identify any progression. The differences in lifetime can best be visualized in trend graphs that plot the mean and standard error of each class for each channel, SI-NADH and WF-NADH, shown in Fig. 4. Although SI-NADH cannot distinguish between mild dysplasia and carcinoma, SI-NADH can differentiate between normal and both mild dysplasia and carcinoma classes. On the other hand, WF-NADH can distinguish carcinoma from mild dysplasia and normal classes, but cannot differentiate normal from premalignancy (mild dysplasia). This difference can most likely be attributed to the effects of epithelial thickening during carcinogenesis and the depth selectivity of the epithelium with structured illumination.

**Table 1:** Summary of statistics for the SI-NADH and WF-NADH lifetime channels.

Null hypothesis	SI-NADH	WF-NADH
Class A = Class B	*p* values	
Mild Dysplasia = Normal	0.001	0.897
Mild Dysplasia = Carcinoma	0.929	0.027
Carcinoma = Normal	0.022	0.034

Null hypothesis	SI-NADH	WF-NADH
	Mean ± Standard Deviation Lifetime Deltas (ns)	
Normal	− 0.01 ± 0.19	0.00 ± 0.12
Mild Dysplasia	− 0.32 ± 0.26	0.03 ± 0.23
Carcinoma	− 0.36 ± 0.29	− 0.31 ± 0.27

*P* values are shown where significance indicates a rejection of the null hypothesis stated in the left column.

Mean lifetime values and standard deviation are displayed based on the verification of approximate normality.

The fluorescence lifetime measurement of NADH is usually obscured by background collagen fluorescence confounding the signal from a normally thin epithelium. Although there are detectable changes in the NADH lifetime during mild dysplasia, they are usually obscured due to overwhelming signal from the collagen layer. Structured illumination addresses this by optically sectioning away the collagen from the epithelium, allowing for the measurement of changes in the NADH lifetime. WF-NADH shows a similar drop in lifetime, but only during the more advanced stages of carcinogenesis. This behavior is clearly shown in Fig. 4A,B. We hypothesize that this is possibly because of the epithelial thickening and invasion into the submucosa that occurs with carcinogenesis^40^. This process both distances the collagen from the image plane in the epithelium and degrades the fluorescence as the epithelial cells invade the submucosa. Because of the morphological changes that occur and the diminishing fluorescence of collagen, the changes in the NADH lifetime are able to be viably measured with WF-NADH in the advanced stages of carcinogenesis. Our data suggest that there is a significant lifetime shift during mild dysplasia that can be measured by SI-NADH, but is only available to WF-NADH during more advanced stages. However, SI-NADH does not have a significant decrease between mild dysplasia and carcinoma. Qualitatively, a decrease can be seen in Fig. 4 (A), but the data is too noisy to make any conclusions. We should also consider the small sample sizes of the classes in the two groups, WF-NADH and SI-NADH, and the variability of the carcinoma class. Additionally, this phenomenon could be unique to our system and induced by a systemic procedure. Other authors’ work indicates that the lifetime should keep decreasing, but, as just mentioned, the variability in our data was too large to draw any statistically significant conclusions^17^. Regardless, Fig. 4A,B succinctly shows the evolution of the lifetime differences as oral mucosa progresses through the stages of carcinogenesis.

To explore the use of SI-FLIM as a diagnostic test, the raw lifetime deltas can be used to generate ROC curves in the task of distinguishing between normal tissue and mild dysplasia. We reduce the complexity of this model by only considering the ROC curve between normal and mild dysplasia for a single feature at a time. This case is of particular interest because it indicates that SI-NADH may be able to better detect malignancy in its early stages of development. The results of both curves are then compared to evaluate which one acts as a better discriminator in this limited case. Figure 4C,D directly compares the ability of SI-NADH to discriminate normal tissue from mild dysplasia against the ability of WF-NADH. The empirical ROC curves are shown plotted alongside their proper binormal fitted models. Because we assume that WF-NADH will perform worse, it should have a lower area under the ROC curve. Knowing this, we can perform a one-tailed t-test between the curves and show that the area under the ROC curve for SI-NADH is statistically larger than the area under the ROC curve for WF-NADH (*p* = 0.031)^41^.

**Figure 5:**
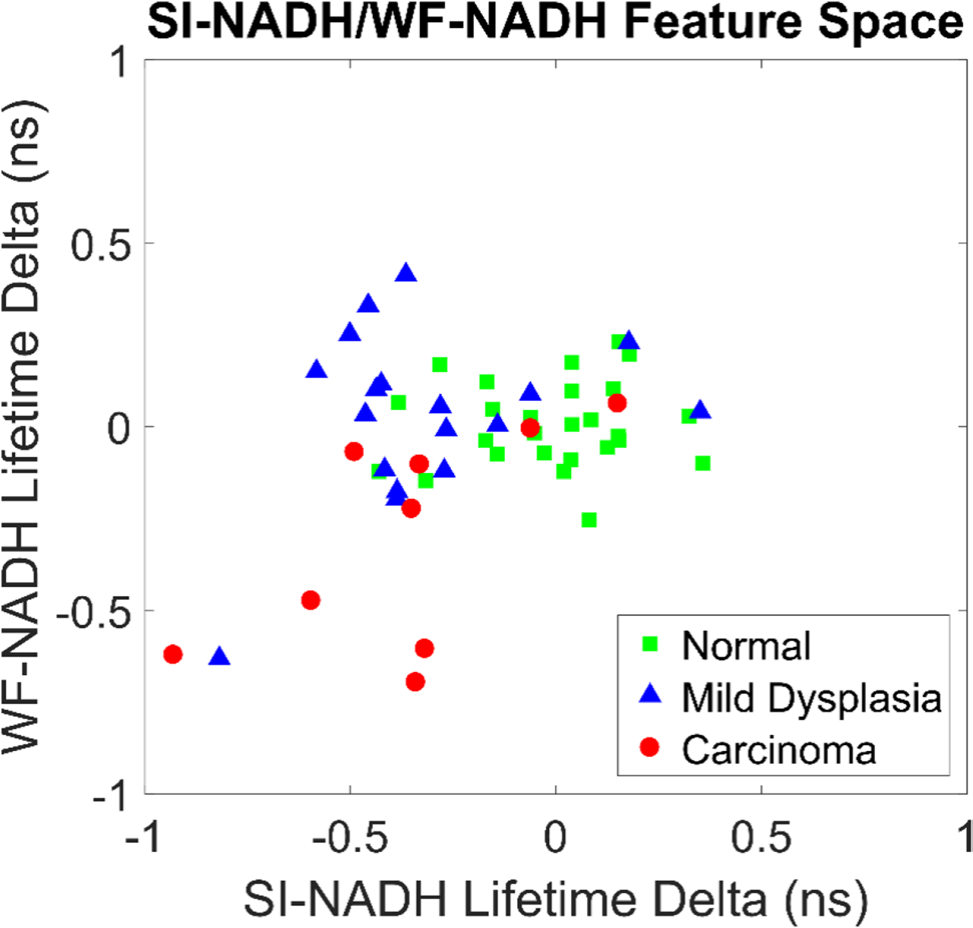
Feature space comparing the relationship between SI-NADH and WF-NADH for three diagnostic cases (normal, mild dysplasia, and carcinoma). Normal resides mostly around a zero delta from itself, as expected. Mild dysplasia shows displacement preferentially along the SI-NADH axis. Carcinoma shows displacement on both axes equally.

Because WF-NADH can be used to distinguish between carcinoma and all other cases, one can easily extrapolate a decision tree to determine which pathology class a given image is in based on the lifetime delta for both WF-NADH and SI-NADH. To help visualize this it is useful to plot the data points in a feature space. Figure 5 shows the two-dimensional feature space with SI-NADH on the lateral axis and WF-NADH on the vertical axis. Three classes, are defined in the legend: normal, mild dysplasia, and carcinoma. The class separations shown in Fig. 4 are readily visible in Fig. 5, but with the added benefit of a more holistic view pertaining to interclass relations. Note that mild dysplasia shows preferential displacement along the SI-NADH axis. This means that the majority of the discrimination power is coming from SI-NADH in this case. For carcinoma, there is approximately equal displacement on both axes, yielding similar discrimination power to both SI-NADH and WF-NADH. A crucial part to note is that SI-NADH does not discriminate well between carcinoma and mild dysplasia while WF-NADH does. Therefore, this results in three clusters contained within the two-dimensional feature space defined by SI-NADH and WF-NADH. With further experiments, and more data, a classifier could be trained to discriminate between the three class clusters.

## Discussion

Although NADH, FAD, and collagen signals were measured with widefield FLIM, the only fluorescence channel measured with both widefield FLIM and SI-FLIM was the NADH channel. This was done to limit the amount of UV exposure and reduce photobleaching of the tissue sample caused by the extended time necessary to acquire SI-FLIM images^31^. Hence, our analysis focused exclusively on the efficacy of SI-NADH as a diagnostic feature against that of WF-NADH. In the future, this method could be easily extended to incorporate SI-FAD as a feature. To do this, the imaging rate would need to be increased so that there is adequate signal for structured illumination when imaging FAD. As of now, much of the signal is reduced due to the extended imaging time and photo bleaching when evaluating NADH. An alternative would be to multiplex imaging so that multiple spectral channels could be imaged at the same time, or use additional excitation sources to preferentially excite FAD^42^.

SI-FLIM was first evaluated by determining its effects on the measurement of the NADH spectral channel. As previously mentioned, widefield FLIM suffers from the integration of fluorescence from out of focus planes. Here, we found that using SI-FLIM on normal tissue to measure the NADH spectral channel decreased the observed fluorescence lifetime value when compared to widefield FLIM, as is shown in Fig. 2. This is the expected behavior that we hypothesized would occur in SI-NADH, as the true NADH fluorescence signal can be reconstructed when the collagen background fluorescence is removed. SI-FLIM preferentially imaged the in-focus content, which is the oral epithelium in this case, and rejected the out of focus light that predominately emanates from the submucosal connective tissue, which is composed primarily of collagen^43^. A paired t-test between SI-NADH and WF-NADH for all normal regions was performed, and the results proved to be statistically significant.

Regions of interest were put into three classes: normal, mild dysplasia, and carcinoma based upon histopathological diagnosis. These classes were then compared using ANOVA, and the results in Table 1 showed that SI-NADH could discriminate between mild dysplasia and normal within our samples, while WF-NADH could not. The efficacy of SI-NADH and WF-NADH in the task of discriminating between mild dysplasia and normal is shown in Fig. 4. It is clear that SI-NADH is the superior discriminator in this sense, an observation which is validated by statistically comparing the two ROC curves. At a fundamental level, this validated our hypothesis and is strong evidence that this pilot study merits a longer follow-up study with more animals. Additional findings of particular interest were that WF-NADH can discriminate between mild dysplasia and carcinoma, and SI-NADH can differentiate between normal and carcinoma better than WF-NADH. These results motivate the addition of structured illumination to WF-FLIM, and show the potential for these modalities to be used in concert to image suspicious lesions. A potential workflow in the clinic could be to first image the lesion with SI-FLIM to assess if a tissue sample is normal/healthy, and then subsequently imaging with WF-FLIM to classify the tissue as mild dysplasia or carcinoma.

As a screening tool for oral cancer, SI-NADH performs well but could be improved. This is apparent when looking at the slowly climbing value of true positive fraction (TPF) with increasing values of false positive fraction (FPF) in Figs. 4 (C),(D). With a TPF of 0.75, the FPF is 0.24; this results in a sensitivity and specificity of 75/76%. Values closer to 85% would be more desirable. A potential explanation for these low sensitivity and specificity values could be the existence of a handful of data points that are considered statistical outliers by definition of being 1.5 times above or below the interquartile range of their respective class. Removing these points from the data set drastically raises the area under the ROC curve for SI-NADH while leaving WF-NADH largely untouched. For example, the area under the empirical ROC curve for SI-NADH is pushed to 0.92 while WF-NADH goes to 0.69. However, due to our small sample size, we did not remove any potential outliers when estimating the sensitivity and specificity.

As a precursor to developing a machine learning classifier, WF-NADH and SI-NADH are plotted in a two-dimensional feature space where the three clinical diagnoses are considered classes. Figure 5 provides a qualitative overview of the interclass and intraclass variation for each diagnosis in terms of WF-NADH and SI-NADH lifetime deltas from normal. Mild dysplasia shows greater spread along the SI-NADH axis than the WF-NADH axis, which signifies greater discrimination power for SI-NADH. This suggests that SI-FLIM outperforms WF-FLIM at detecting lesions earlier in the disease progression, which is paramount to the treatment and survival of oral cancer. One shortcoming of this study is that the carcinoma class is highly varied and possesses the lowest sample number. Therefore, a classifier to distinguish between all three classes was not considered feasible for this limited dataset.

Although our analysis yielded promising results, we believe that they could have been enhanced by certain improvements to the system. To start, the measured lifetime features consisted of the median lifetime values for a given field of view of ~ 0.9 mm diameter. In vivo FLIM for diagnostic purposes is typically performed over a much larger field of view, ~ 2–10 mm diameter^44,45^. This much smaller field of view could have contributed to completely missing entire diagnosed regions. In the methods section, we defined an imaging grid that contained twelve regions, three lines with four quadrants per line, in an approximately 16 mm × 16 mm area. The position of the imaging lines relative to the histology tissue section is fairly certain; however, each quadrant occupies a 4 mm section with one 0.9 mm field of view taken in it. Another factor that influences co-registration is tissue shrinkage and damage during fixation and histopathology. Because of this, images that were directly adjacent to the diagnosed quadrant were considered to possess the same diagnoses. This could have potentially produced regions of interest that were not co-registered with the histopathological diagnoses they were assigned. One place where this is evident is in Fig. 5. A handful of carcinoma diagnoses appear firmly center of the cluster of normal diagnoses. However, the uncertainty in our image placement within the histopathologic sections leaves us unable to reject them from the analysis. With better co-registration, by either increasing the field of view or increasing the imaging speed to acquire more fields of view, this would be less of an issue. The three-phase SIM technique was the major limitation to our imaging speed, but new advances that only require one structured image could alleviate this problem^46^. Poor contrast of the illumination pattern in the epithelium also presented a challenge. Our experiment used an air objective, leaving the oral epithelium exposed to the environment. What appeared to be secretions from the epithelium reduced our ability to resolve the structured illumination pattern and required drying the tissue via compressed air prior to imaging. This could be solved in future experiments by implementing a water immersion physiology lens, but this may not be practical for translation to clinical use.

In summary, we have proposed and demonstrated structured illumination fluorescence lifetime imaging microscopy as a potential screening tool for oral cancer. A statistically significant difference was found between the fluorescence lifetime values estimated by SI-FLIM and WF-FLIM, suggesting the structured illumination can be added to WF-FLIM to acquire the true NADH fluorescence lifetime and achieve a more accurate metabolic signature of the sample. SI-FLIM was also able to better discriminate normal from dysplastic or pre-malignant tissue samples than WF-FLIM; this would help improve the rates of early detection of oral cancer and lead to more positive prognoses. SI-FLIM has the clinical potential to be a useful discriminator between the early stages of pre-malignancy (mild dysplasia) and normal tissue. This method has the potential to lower the time delay between the onset, the detection, and the subsequent treatment of oral squamous cell carcinomas.
